# Shell-Isolated Nanoparticle-Enhanced Raman Spectroscopy for Probing Riboflavin on Graphene

**DOI:** 10.3390/ma15051636

**Published:** 2022-02-22

**Authors:** Agnė Zdaniauskienė, Ilja Ignatjev, Tatjana Charkova, Martynas Talaikis, Algimantas Lukša, Arūnas Šetkus, Gediminas Niaura

**Affiliations:** 1 Department of Organic Chemistry, Center for Physical Sciences and Technology (FTMC), LT-10257 Vilnius, Lithuania; agne.zdaniauskiene@ftmc.lt (A.Z.); ilja.ignatjev@ftmc.lt (I.I.); tatjana.charkova@ftmc.lt (T.C.); martynas.talaikis@gmc.vu.lt (M.T.); 2 Department of Physical Technologies, Center for Physical Sciences and Technology (FTMC), LT-10257 Vilnius, Lithuania; algimantas.luksa@ftmc.lt (A.L.); arunas.setkus@ftmc.lt (A.Š.)

**Keywords:** SHINERS, GERS, DFT, riboflavin, ribityl, graphene, Raman

## Abstract

Graphene research and technology development requires to reveal adsorption processes and understand how the defects change the physicochemical properties of the graphene-based systems. In this study, shell-isolated nanoparticle-enhanced Raman spectroscopy (SHINERS) and graphene-enhanced Raman spectroscopy (GERS) coupled with density functional theory (DFT) modeling were applied for probing the structure of riboflavin adsorbed on single-layer graphene substrate grown on copper. Intense and detailed vibrational signatures of the adsorbed riboflavin were revealed by SHINERS method. Based on DFT modeling and detected downshift of prominent riboflavin band at 1349 cm^−1^ comparing with the solution Raman spectrum, π-stacking interaction between the adsorbate and graphene was confirmed. Different spectral patterns from graphene-riboflavin surface were revealed by SHINERS and GERS techniques. Contrary to GERS method, SHINERS spectra revealed not only ring stretching bands but also vibrational features associated with ribityl group of riboflavin and D-band of graphene. Based on DFT modeling it was suggested that activation of D-band took place due to riboflavin induced tilt and distortion of graphene plane. The ability to explore local perturbations by the SHINERS method was highlighted. We demonstrated that SHINERS spectroscopy has a great potential to probe adsorbed molecules at graphene.

## 1. Introduction

Electronic properties and function of graphene depend on the surface structure, origin of the defects [1,2], doping type and nature of dopants [3,4,5,6], and adsorption of molecules and ions at the surface [7,8]. Adsorbed molecules may modify the carrier mobility of underlying graphene layer [8]. In the case of uncontrolled adsorption it is difficult to construct electronic systems with predictable and reliable properties. Graphene science and development of graphene-based technologies require to control adsorption processes and understanding how the defects and impurities modify physico-chemical properties of these systems at molecular level [9]. It was revealed that adsorbed aromatic molecules on single layer graphene modulate Fermi energy [10]. Furthermore, in order to increase the lithium storage capacity of graphene nanosheets in rechargeable lithium-ion batteries, it is necessary to modify the structure of the graphene [11]. Therefore, understanding the surface and interface chemistry of graphene-based systems is an important topic in physical chemistry and material science.

Raman spectroscopy is a non-destructive, sensitive, and powerful technique able to provide detailed structural information about various carbon nanostructures [12,13]. In addition, resonance Raman investigations can afford important information about the electronic structure of the material [14]. Because of extended π-electron system of graphene and graphene oxide, excitation in the visible spectral region affords resonantly enhanced Raman spectra [12,14]. Nevertheless, resonance Raman spectroscopy provides information on the structure of graphene skeleton [12]; usually, no direct information on the structure of molecular groups covalently attached to the carbon matrix or adsorbed compounds can be acquired. In some cases, adsorbed organic molecules can be probed by graphene-enhanced Raman spectroscopy (GERS) [15,16,17,18,19,20,21]. In GERS the dominant Raman signal enhancement mechanism is chemical enhancement due to charge transfer excitation [17,21]. Thus, the enhancement strongly depends on (i) appropriate energy levels of adsorbate and (ii) the structure of the molecule [17]. Flat molecules possessing D_nh_ symmetry are favorable because of better compatibility with graphene structure [17]. Being dependent on chemical enhancement mechanism, GERS demonstrate distinctive molecular selectivity [16].

To observe functional groups of graphene oxide, adsorbed impurities or purposely-adsorbed compounds on graphene, the alternative spectroscopic technique is required. Recently, Tian et al. approached a novel surface-enhanced Raman spectroscopy (SERS) technique and called it “shell-isolated nanoparticle-enhanced Raman spectroscopy” (SHINERS) [22]. The method is based on the enhancement of Raman signal by strong electromagnetic field provided by metallic core nanoparticles surrounded by a few nanometer thick dielectric shell [22,23,24,25,26,27,28]. Dielectric shell-covered nanoparticles have significant advantages over bare nanoparticles. The inert shell prevents the interaction between the metal core and the system. Moreover, the shell protects the core from aggregation, oxidation, and also from contaminating the system under study. SHINERS has been used to obtain molecular-level knowledge of a diverse set of surfaces, from adsorbates on flat meta-single-crystals to semiconductors and from food samples to spectroscopic analysis of living cells and detection of circulating tumor cells in blood [23,24,25,26,27,28,29,30,31,32,33,34,35].

**Figure 1:**
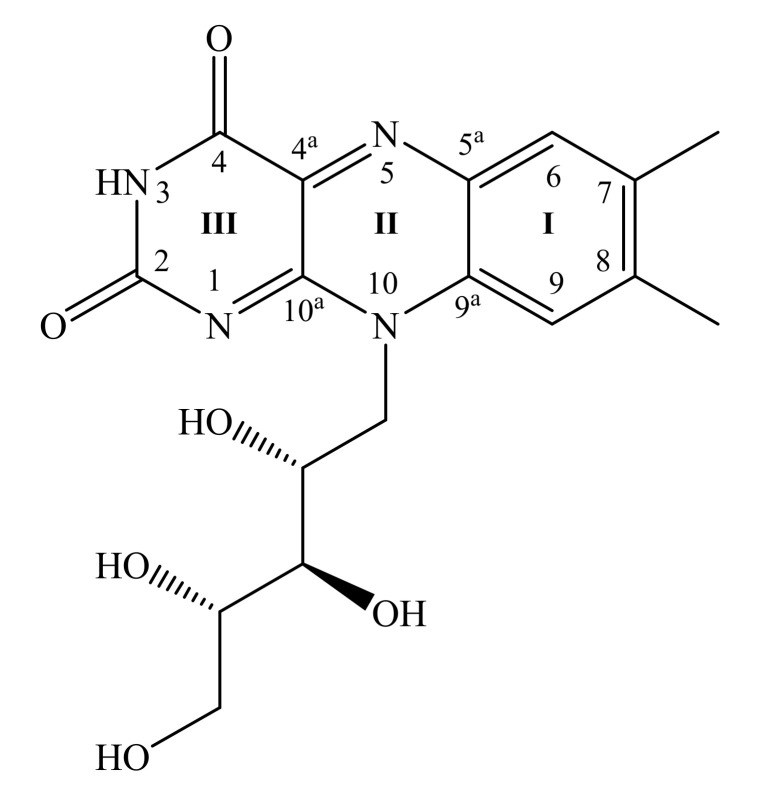
Riboflavin molecular structure and numbering of isoalloxazine ring atoms.

Riboflavin (Rf) or vitamin B_2_ is a water-soluble vitamin that plays an essential role in cellular biochemistry [36]. Renewed interest in its monitoring and investigation of molecular interactions stems from the important role of Rf in the prevention of health diseases like migraine, cancer, hypertension, and chronic diseases associated with oxidative stress [37]. Rf can be employed in electrochemistry for different purposes as well as it can be detected electrochemically due to its aromatic nature (Figure 1). Previously, we investigated electropolymerisation of riboflavin in diverse media [38]. Riboflavin films were characterized spectroscopically, microscopically, and electrochemically. Promising sensors may be constructed from hybrid Rf-graphene conjugates. For a molecular-level understanding of the bonding and structure of adsorbed Rf, vibration spectroscopy seems to be very promising. However, it was hard to research adsorbed Rf onto highly oriented pyrolytic graphite by Raman spectroscopy [38]. Thus, surface-enhanced Raman spectroscopy methods should be applied.

The main aim of our work was to demonstrate the applicability of the SHINERS technique to acquire molecular-level information for adsorbed Rf on the graphene surfaces. The detailed insights into the interaction of Rf and graphene are provided from the analysis of coupled SHINERS, GERS, and DFT modeling approaches.

## 2. Materials and Methods

### 2.1. Materials

All used reagents and solvents (Merck, Darmstadt, Germany) were used without further purifications: gold (III) chloride trihydrate (HAuCl_4_ · 3H_2_O, 99%), trisodium citrate dihydrate (HOC(COONa)(CH_2_COONa)_2_ · 2H_2_O, 99%), (3-aminopropyl)trimethoxysilane (H_2_N(CH_2_)_3_Si(OCH_3_)_3_, APTMS, 97%), sodium silicate solution (10% NaOH, 27% SiO_2_). Graphene substrate was received from Graphenea (Donostia-San Sebastian, Spain), and riboflavin was obtained from Alfa Aesar (Ward Hill, MA, USA). All solutions were prepared with ultrapure water (resistivity of 18.2 MΩ cm) from Direct-Q 3UV (Merck, Darmstadt, Germany) water purification system.

### 2.2. Synthesis of Silicon Dioxide Covered Spherical Gold Nanoparticles

Initially, 50 mL of an aqueous HAuCl_4_ solution (0.01 wt%) was brought into a round-bottom flask and boiled in an oil bath (150 °C) with vigorous stirring. The solution reached boiling temperature in 3 min. Then 0.35 mL of sodium citrate (1 wt%) were quickly added into the boiling solution and the mixture was refluxed for 30 min. Next, the solution was left to attain room temperature (about 2 h); so the red colloid of gold nanoparticles (Au NPs) was obtained. For the growth of an ultrathin layer of silicon dioxide over nanoparticles, 0.6 mL APTMS (1 mM) was added to 45 mL of Au NPs. The mixture was vigorously stirred for 15 min; then 4.8 mL of sodium silicate solution (0.54% wt/wt) was added and the mixture was stirred again for 3 min at room temperature. The flask was placed in an 85–90 °C water bath and stirred for 45 min. Then, to stop the reaction process, the flask was placed in the ice bath (3 °C) for 30 min. The cold mixture was transferred into several 1.5 mL test tubes and centrifugated at 5500 rpm for 15 min. After that, the supernatant was removed, the 1.5 mL of water was added, and nanoparticles were dispersed again. Next, tubes were centrifuged for another 15 min and supernatant was removed again to obtain Au@SiO_2_ nanoparticles as a concentrated colloidal suspension. Therefore, cleaned Au@SiO_2_ nanoparticles were kept in the dark at low temperature (5 °C) until were used in SHINERS experiments.

### 2.3. High-Resolution Transmission Electron Microscopy (HR-TEM)

High-resolution transmission electron microscopy (HR-TEM) image was recorded by Tecnai G2 F20 X-TWIN microscope (FEI, Eindhoven, The Netherlands) equipped with scanning TEM module and high-angle annular dark-field detector for Z-contrast imaging. FEI Helios Nanolab 650 dual beam microscope with an Omniprobe manipulator (FEI, Eindhoven, The Netherlands) was employed to prepare specimens for the measurement.

### 2.4. Riboflavin Adsorption

Commercially available graphene substrate grown onto Cu foil was washed with water. Chemisorbed riboflavin layer onto graphene substrate was formed in 1 mM riboflavin water solution for 14 h. Later it was washed with ultrapure water and dried at room temperature in a fume hood for about 30 min. For SHINERS experiments, 5 µL of concentrated Au@SiO_2_ solution were dropped onto pure graphene and adsorbed riboflavin onto graphene substrate surfaces and dried in a fume hood for about 30 min.

### 2.5. Raman Measurements

The GERS and SHINERS spectra were recorded by inVia Raman (Renishaw, Wotton-under Edge, UK) spectrometer equipped with thermoelectrically cooled to −70 °C CCD camera and a confocal Leica microscope. Two different wavelengths, 785 and 532 nm laser beams were used as the excitation source. The 180 °C scattering geometry was employed in Raman spectroscopy studies. The 50×/0.75 NA objective lens and 1200 lines/mm (for 785 nm) and 1800 lines/mm (for 532 nm) gratings were exploited to record the Raman spectra. Laser power at the sample surface was restricted to 0.9 and 0.6 mW for 785 and 532 nm excitations, respectively. Raman scattering wavenumber axis was calibrated by using the silicon peak at 520.7 cm^−1^ or by recording the polystyrene standard (ASTM E 1840) Raman spectrum. Raman spectra were corrected by polynomial function background subtraction. The GERS spectra were recorded by using 532 nm excitation wavelength, while 785 nm wavelength was applied for acquisition the SHINERS spectra. Integration time was 10 s and each spectrum was recorded by accumulation of 10 scans yielding total 100 s collection time. Both SHINERS and GERS spectra were normalized to 1 s intensity. No smoothing procedures were applied to the experimental data.

Raman spectra of riboflavin powder and riboflavin solution were recorded by using HyperFlux PRO Plus (Tornado Spectral Systems, Mississauga, ON, Canada) spectrometer at 785 nm excitation. Laser powers were 15 mW for Rf powder and 495 mW for Rf-water solution of approximately 0.3 mM. The overall accumulation time was 37.5 and 1375 s for Rf powder and Rf-water solution spectra, respectively. For excitation and collection of the Raman spectra, the fiber-optic cable was employed. The laser beam was focused to a 100 μm diameter spot. The calibration of the wavenumber axis was confirmed with the polystyrene standard (ASTM E 1840) spectrum.

Parameters of the vibrational bands were determined by fitting the experimental spectra with Gaussian-Lorentzian shape components using GRAMS/A1 8.0 software (Thermo Scientific, Waltham, MA, USA).

### 2.6. Theoretical Modeling

Molecular interactions of hydrogen-terminated graphene nanosheet C_54_H_18_ and riboflavin in the gas phase were modeled using Orca 5.0.1 software [39]. We used B3LYP functional with split-valence Pople and all-electron Karlsruhe basis sets. It is known that DFT theory inaccurately describes weak interactions, therefore the Becke-Johnson damping method (D3BJ) was included in computations to correct for missing dispersion forces. Molecular optimization was done and energies were calculated using Pople’s 6-311G(d,p) basis set; to complement the results separate molecular optimization was carried out at def2-SVP basis set level followed by the estimation of electronic properties using the def2-TZVP. The adsorption energy *E*_ad_ was calculated according to the relation:

(1) $$E_{ad}=E_{G-Rf}-\left(E_{G}+E_{Rf}\right)-\partial_{BSSE}$$

where *E*_G-Rf_, *E*_G_, and *E*_Rf_ are energies for graphene-riboflavin complex, graphene, and riboflavin. The basis set superposition error (BSSE) was corrected by a counterpoise method that introduces ∂_BSSE_ to Equation (1) [40]. Vibrational spectra were obtained at B3LYP/6-31G(d) after the geometry optimization at the same level. Theoretical spectra include (1) Rf in gas phase, (2) Rf in water simulated by conductor-like polarizable continuum model (CPCM), and (3) Rf in complex with graphene (G). The (3) was achieved by full optimization of G-Rf complex followed by freezing the G and numerical calculation of Rf frequencies. All the vibrational spectra were completed with no imaginary frequencies. The intensities and frequencies were scaled [41]. Total density of states (TDOS) and partial density of states (PDOS) [42] were calculated using Multiwfn 3.8 software (2021, Beijing Kein Research Center for Natural Sciences, Beijing, China) by means of the SCPA method [43]. Visualizations were made using ChemCraft 1.8 software (2021, Grigoriy A. Andrienko, Ivanovo, Russia) [44].

## 3. Results and Discussion

### 3.1. GERS Spectra of Riboflavin on Graphene

**Figure 2:**
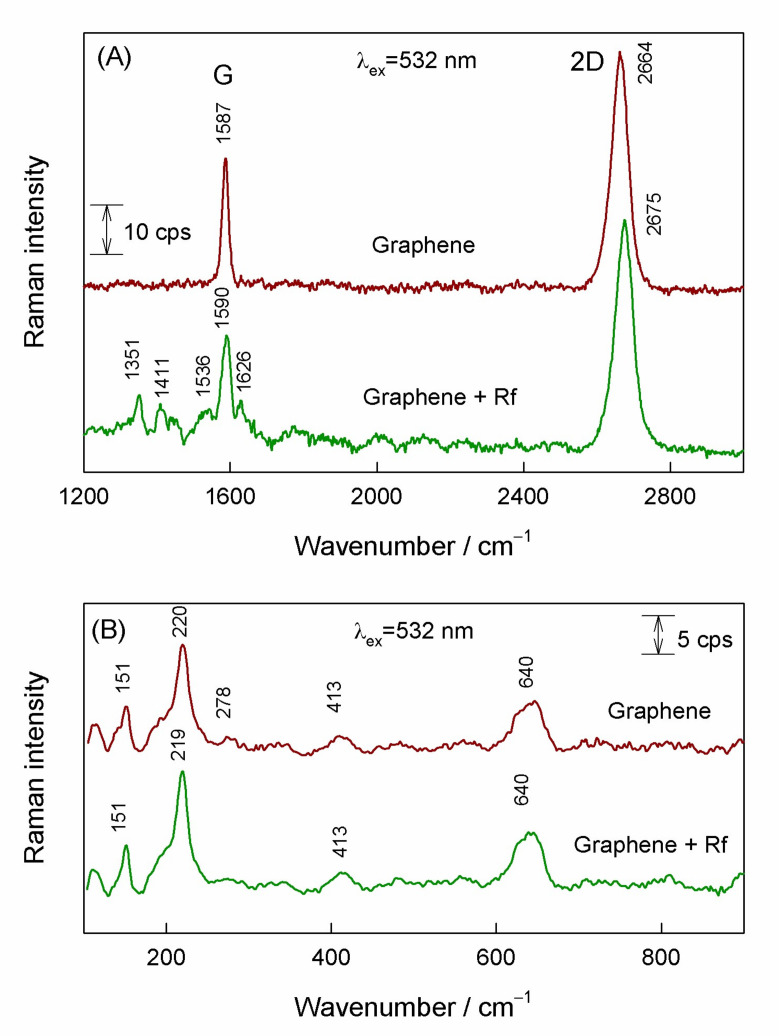
(**A**) Resonance Raman spectra of graphene and graphene with adsorbed riboflavin excited at 532 nm wavelength in the spectral region of 1200–2800 cm^−1^, and (**B**) in the low frequency spectral region of 100–900 cm^−1^.

Figure 2 compares 532-nm excited resonance Raman spectra of graphene on a copper substrate before treatment with riboflavin solution and after the riboflavin adsorption. In the spectral region from 1200 to 3000 cm^−1^ (Figure 2a) the dominant feature at 2664 cm^−1^ visible in the spectrum of the substrate before treatment with riboflavin solution is associated with prominent 2D-band of graphene structure [12,45,46]; while the G-band appears at 1587 cm^−1^. Both 2D and G-bands are always allowed in the resonance Raman spectra. No D-band associated with presence of defects is visible in the vicinity of 1350 cm^−1^. The intensity ratio I(2D)/I(G) can be used for the evaluation of the number of graphene layers [45]. In the case of the monolayer, the I(2D)/I(G) > 1 and the 2D band must be fitted by using one Lorentzian form component [14]. For our studied sample, we found I(2D)/I(G) = 1.8 and Lorentzian form shape of 2D band. Thus, resonance Raman spectroscopic analysis of substrate sample before riboflavin adsorption revealed presence of high quality (without manifestation of defects) single-layer graphene. Low-frequency Raman spectrum (Figure 2B) exhibits several well-defined bands at 151, 220, and 640 cm^−1^ which are characteristic to Cu_2_O compound [47].

Adsorption of riboflavin induced several clear changes in the high-frequency spectrum (Figure 2a); both characteristic graphene bands were found to be blue shifted and new low-intensity bands became visible in the spectral region 1300–1650 cm^−1^. Shift of G and 2D bands indicates riboflavin-induced doping of graphene layer. It should be noted that the relative intensity of graphene bands remained similar as before adsorption (I(2D)/I(G) = 1.92). The new bands located at 1351, 1411, and 1536 cm^−1^ are characteristic for riboflavin ring [38] and indicate the Raman scattering enhancement by graphene substrate (GERS). Low frequency spectral region reveals no riboflavin-induced changes in Cu_2_O Raman spectrum (Figure 2b). Thus, excitation at 532 nm provides the possibility to detect adsorbed riboflavin through the GERS mechanism, but the intensity of the adsorbate bands was found to be very low. More detailed analysis of riboflavin interaction with graphene substrate will be given later on in this manuscript.

### 3.2. SHINERS Spectra of Riboflavin on Graphene

**Figure 3:**
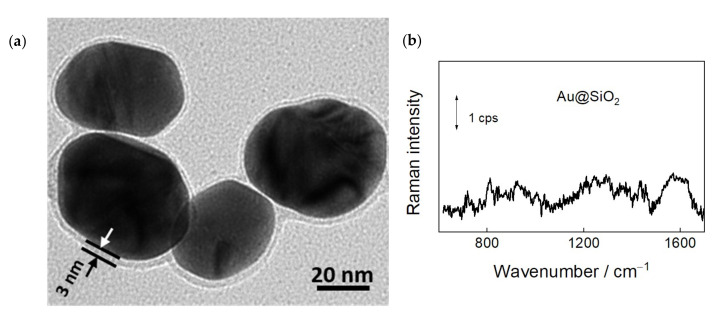
(**a**) High-resolution transmission electron microscopy (HR-TEM) image of Au@SiO_2_ (50 ± 5 nm) nanoparticles employed in SHINERS experiments; (**b**) SHINERS spectrum of bare Au@SiO_2_ nanoparticles dispersed on a steel substrate.

To enhance vibrational spectrum of adsorbates on graphene, we have employed SHINERS approach. For this technique, we have prepared highly effective and stable plasmonic nanoparticles with gold core and silica shell, Au@SiO_2_. The high-resolution transmission electron microscopy image of Au@SiO_2_ nanoparticles is shown in Figure 3a. Image exhibits a dark gold core surrounded by a brighter SiO_2_ shell. In SHINERS spectroscopy the parameters of gold core determine the efficiency of plasmonic surface enhancement, while the dielectric shell must prevent from direct interaction of metal with adsorbates and protect from degradation [22,23,24]. Thus, the shell must be sufficiently thick to eliminate pinholes and sufficiently thin to maintain the high surface enhancement provided by the plasmonic core. Figure 3a shows that the thickness of SiO_2_ shell was about 3 nm. The size of nanoparticles was found to be 50 ± 5 nm. These nanoparticles previously were used for the analysis of self-assembled monolayers on a smooth gold substrate at the electrochemical interface [30] and for spectroscopic characterization of living yeast cells [29].

Because gold nanoparticles support the plasmonic enhancement of electric field in the red and NIR spectral region, we employed 785 nm laser line radiation for excitation of SHINERS spectra. One can see that the bare Au@SiO_2_ nanoparticles exhibit low intensity spectral features (Figure 3b). Observed bands may be associated with the adsorbed compounds from the preparation of nanoparticles.

**Figure 4:**
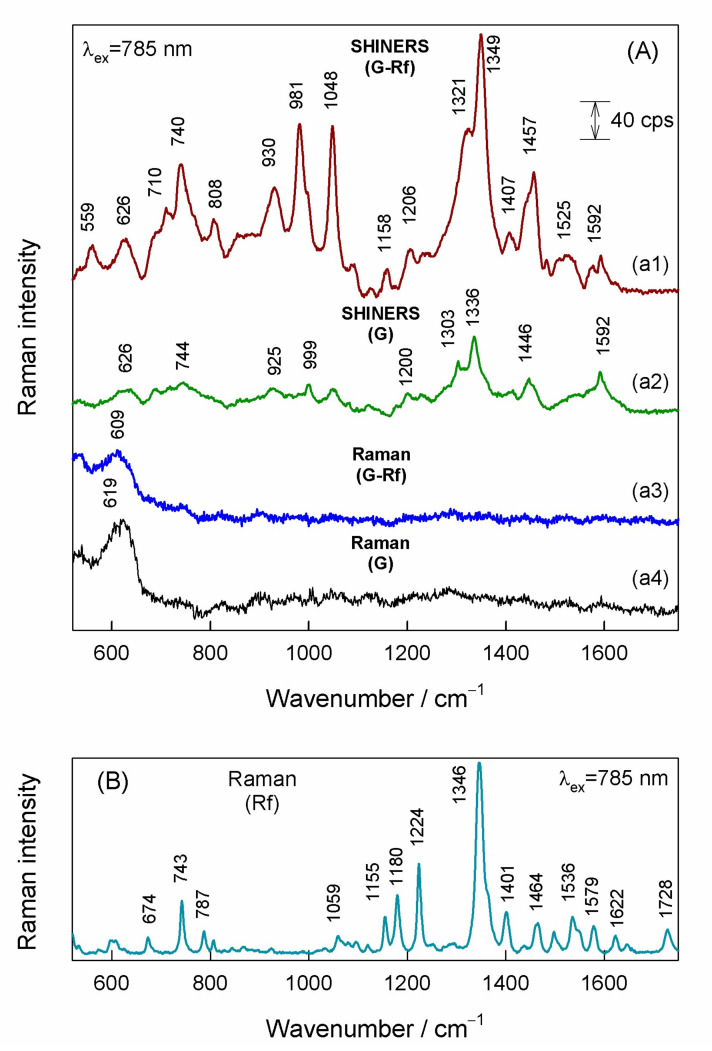
(**A**) SHINERS spectra of (**a1**) riboflavin adsorbed onto graphene with Au@SiO_2_ nanoparticles, and (**a2**) pure graphene substrate with Au@SiO_2_ nanoparticles, and Raman spectra of (**a3**) riboflavin adsorbed onto graphene without nanoparticles, and (**a4**) pure graphene substrate without nanoparticles. The luminescence background in spectra (**a3**) and (**a4**) was subtracted by using polynomial function fit. Spectra are shifted vertically for clarity. (**B**) Raman spectrum of riboflavin powder. Excitation wavelength is 785 nm.

Figure 4A compares SHINERS spectra from the graphene with adsorbed riboflavin (G-Rf) and the graphene substrate before the adsorption (G) with the corresponding Raman spectra observed without the use of the Au@SiO_2_ nanoparticles. Only broad band centered near 609–619 cm^−1^ was visible in the Raman spectra of the graphene and the graphene-riboflavin samples. This band evinces the presence of Cu_2_O oxide under the graphene layer. Spreading of the Au@SiO_2_ nanoparticles over the graphene substrate results in SHINERS spectrum with several clearly-defined bands (Figure 4(a2)). The 1592-cm^−1^ feature was assigned to G-band of graphene monolayer, while the bands in the vicinity of 1303–1336 cm^−1^ might be related with graphene D-band activated by the presence of defects in the vicinity of Au@SiO_2_ nanoparticles. Other low intensity bands most likely originate from adsorbed impurities on graphene surface and/or covalently bonded functional groups at graphene plane and impurities from Au@SiO_2_ nanoparticles itself. The 1446-cm^−1^ feature falls in the characteristic spectral region of scissoring deformation vibration of CH_2_ groups. Different SHINERS spectrum was observed after spreading the Au@SiO_2_ nanoparticles on the graphene surface with adsorbed riboflavin (Figure 4(a1)). The most intense band in the spectrum centered at 1349 cm^−1^ can be immediately assigned to riboflavin-related mode by comparing with the Raman spectrum of riboflavin powder (Figure 4B). This band is associated mainly with symmetric stretching ν_s_(C2-N3-C4) + stretching of rings I, II, III vibrational mode [38,48,49,50]. Other riboflavin ring bands are clearly visible at 710, 740, 1158, 1407, 1457, and 1525 cm^−1^. The band at 1525 cm^−1^ corresponds to stretching vibration of rings I, II, and III, and the 1457 cm^−1^ band to CH_3_ deformation vibration coupled stretching vibrations of rings I and II and ribityl bending mode. The 740 cm^−1^ and 710 cm^−1^ modes are ring I in-plane bending and rings I, II, and III in-plane deformation vibrations, respectively. The low-intensity band 1592 cm^−1^ is associated with graphene G-band vibrational mode. The origin of intense bands at 930, 981, and 1048 cm^−1^ is not completely clear; these bands might be related with stretching vibration of ribityl chain. More detailed analysis of spectroscopy of adsorbed riboflavin and effects of interaction with graphene substrate is provided below.

### 3.3. DFT Modelling Predicts Graphene-Riboflavin Interactions

**Figure 5:**
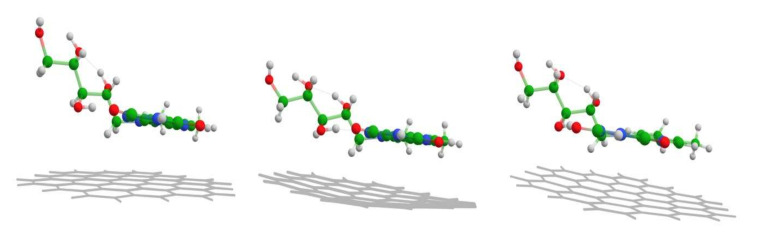
1st, 15th, and 73rd (converged) geometry optimization steps at B3LYP/6-311G(d,p) theory level. Differences in molecular design are merely for convenience to the eye.

The energy minimization moves riboflavin into the orientation parallel to the graphene nanosheet, with an average 3.3 Å distance between the polycyclic Flavin moiety and the graphene (Table 1). The distance is similar to the ones predicted for similar compounds adsorbed to the graphene [51,52]. The parallel stacked configuration enables the π electron contact of graphene and riboflavin molecules and in such a way facilitates charge transfer (CT), which is considered the main mechanism for GERS [16,21]. The ribityl part of the Rf molecule during the energy minimization also comes into closer contact with the graphene plane, while the graphene slightly tilts and distorts from its initial flat shape (Figure 5).

Here, we studied interactions in the gas phase. Calculations predict sufficiently negative adsorption energy *E*_ad_ of −22.39 for B3LYP/6-311G(d,p) and −33.35 kcal/mol for B3LYP/def2-TZVP basis sets. Generally, adsorption energy can be broken down into physically meaningful stabilizing components: electrostatic, dispersion, polarization, and charge transfer energies, and the stabilization components: Pauli repulsion and preparation energies [51]. The dispersion energy may be attributed to the overlap between the π orbitals of G and Rf. Furthermore, electron charge transfer (CT) is another important factor that facilitates adsorption. Hirshfeld population analysis provides a more robust and basis set-independent CT measure compared with Mulliken population analysis [51,53]. Hirshfeld analysis indicated the formation of donor-acceptor pair through the π-π overlay with a transfer of 0.12e from G to Rf that also adds to the net attractive interaction between the two molecules. These findings are in line with the chemical mechanism of GERS, where electronic coupling between the graphene and adsorbent through the mixing of molecular orbitals and charge transfer is required for enhancement to occur [16,54,55]. For a molecule to undergo GERS-enhancement molecular structure and energy levels have to comply with selection rules [16]. First, graphene’s Fermi level must lie within adsorbate’s HOMO-LOMO gap, and second, the symmetries of two molecules have to agree.

**Figure 6:**
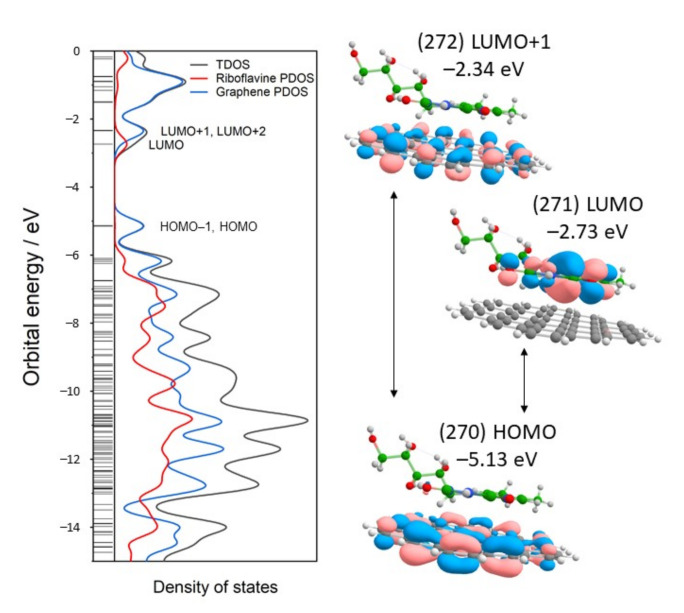
Total and partial density states (TDOS and PDOS) of the graphene-riboflavin complex (**left** panel). Thin horizontal lines indicate molecular orbitals (MOs), while thicker lines—degenerate MOs. HOMO, LUMO, and LUMO+1 are molecular orbitals with their energies indicated (**right** panel). Calculated at B3LYP/6-311G(d,p) theory level.

To investigate the electronic complex structure, density of states (DOS) plots were constructed. DOS shows the distribution of energy levels that electrons can occupy at a given molecule. DOS could be plotted as total DOS (TDOS) for the full complex and as a partial DOS (PDOS) for individual components of the complex. Figure 6 shows TDOS for the G-Rf complex and PDOS for the G and Rf fragments. From PDOS, the degenerate π-orbital of HOMO and HOMO-1 states are localized at graphene near −5.13 eV, while the LUMO occupy riboflavin Flavin group at −2.73 eV. LUMO+1 and LUMO+2 are graphene’s degenerate orbitals near −2.34 eV. The energy of the frontier orbitals provides a measure for ionization potentials (related to *E*_HOMO_) and electron affinity (related to *E*_LUMO_). The energy difference between HOMO and LUMO is regarded as the fundamental energy gap *E*_fund_, which quantitatively determines chemical potential (μ), molecular hardness/softness, and electrophilicity [56]. The smaller *E*_fund_ signifies the higher molecular reactivity and lower kinetic stability. The predicted *E*_fund_ for the molecular complexes is 2.39 eV at 6-311G(d,p) and 2.31 eV at def2-TZVP basis sets calculations. These values are smaller compared to those of Rf optimized in the gas phase.

TD-DFT at B3LYP/6-311G(d,p) was used to calculate the optical gap energies *E*_opt_ of the G-Rf complex (Table 2). Usually, the *E*_opt_ is considerably smaller compared to the *E*_fund_ [57]. TD-DFT predicts several vertical excitations in a visible region for Rf in a gas phase. A very weak transition at 608 nm (f = 0.0003) and stronger at 457 nm (f = 0.1900) ascribed to HOMO→LUMO and HOMO-1→LUMO excitations. For the G-Rf complex, the first transition occurs at 651 nm (f = 0.0015), followed by transition at 639, 433, and 428 nm. These transitions start at different orbitals in the 267−270 range that are mainly located at graphene nanosheet and are targeted to the 271st molecular orbital located at Flavin group. Compared to the sole Rf, the introduction of graphene perturbs its electronic structure that shits absorption to the red and increases the transition probability.

**Figure 7:**
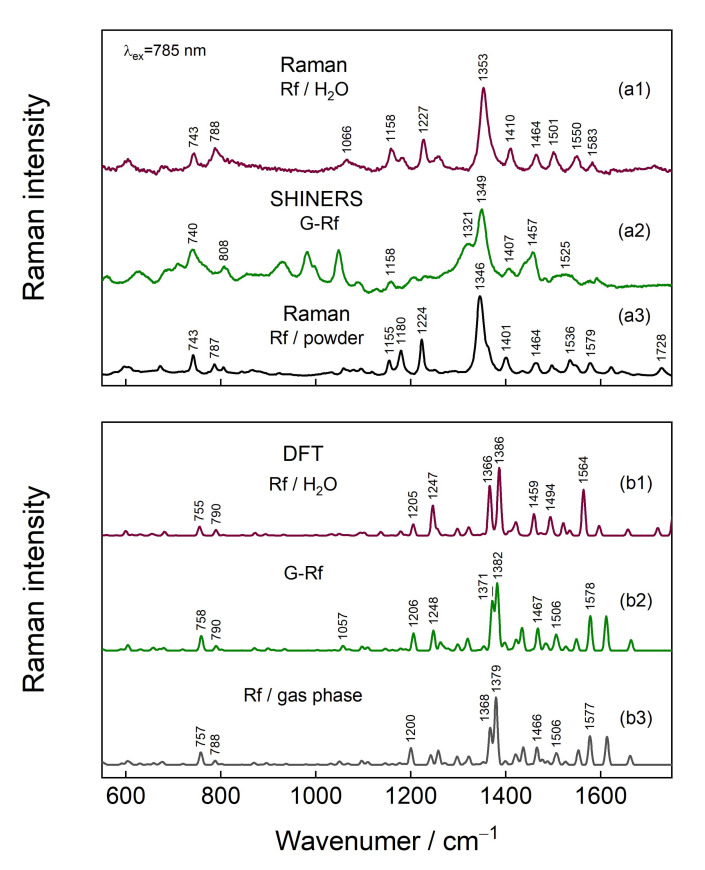
(**a1**) Raman spectra of Rf solution, (**a2**) G-Rf GERS-SHINERS spectrum and (**a3**) Rf powder measured with 785 nm excitation. (**b1**) DFT calculated spectra at 6-31G(d) level of Rf optimized in implicit water modeled using CPCM, (**b2**) G-Rf complex in a gas phase, and (**b3**) Rf in a gas phase.

Figure 7 compares experimental and calculated Raman spectra of Rf in different environments. The dominant vibration at 1345–1353 cm^−1^ is assigned to the ν_s_(C2-N3-C4) + stretching motion of rings I, II, and III (atom numeration in Figure 1) [38,48,49,50,58]. In fact, DFT predicts several vibrational modes that are related to the ring stretching in a 1365–1380 cm^−1^ spectral region. However, there is a difference between the intensity patterns of experimental and theoretical spectra, which might be assigned to a relatively inexpensive basis set (6-31G(d)) used for modeling. A clear 4 cm^−1^ redshift, which is also supported by DFT, is observed for the Rf transfer from aqueous solution to graphene surface. Such a frequency downshift of the vibrational modes of atoms involved in delocalized π orbitals has been previously linked to π-stacking [58,59], therefore, it is clear experimental evidence for direct Rf and G interaction. The π-stacking is supposed for crystal structure bearing Rf as well. The band in the Rf-crystal spectrum is downshifted by 8 cm^−1^ compared to the one in the solution spectrum. Comparing experimental spectra obtained from Rf in different environments, the bands in the experimental G-Rf spectrum at 1158, 808, and 740 cm^−1^ are assigned to Rf. However, DFT modeling does not predict strong bands in the 900–1000 cm^−1^ spectral region.

### 3.4. Comparison of SHINERS and GERS

SHINERS and GERS spectroscopies exhibit very different spectral patterns from graphen-riboflavin surface. The main observed vibrational features and their assignments are listed in Table 3. GERS spectra were excited with 532 nm wavelength and the enhancement mechanism is associated with the CT excitations. Therefore, only Rf ring vibrational modes of atoms involved in π-electron interaction are enhanced. In contrast, SHINERS spectroscopy based on plasmon-enhancement mechanism provides detailed information not only on the Rf ring vibrational modes but also some vibrations of ribityl chain became visible. In addition, the normalized to laser power relative intensity of the prominent 1349 cm^−1^ band of Rf was found to increase by a factor of 26 in SHINERS spectrum comparing with GERS. In the fingerprint spectral region SHINERS spectrum exhibited three well-defined bands (1048, 981, and 930 cm^−1^), which were tentatively assigned to vibrational modes having a high contribution from vibration of atoms in ribityl chain (Table 3). Infrared spectrum of riboflavin shows intense bands in the frequency region from 1100 to 1000 cm^−1^ due to vibration of ribityl group [58]. In solution, Raman spectrum intensity of these bands was found to be very low; only the high frequency band was visible near 1066 cm^−1^. High downshift in frequency suggests the involvement of ribityl chain of riboflavin in interaction with graphene surface. An intriguing observation in SHINERS spectrum is the intense and broad feature near 1321 cm^−1^. Such band was not observed nor in solution or powder spectra of Rf (Figure 7). We tentatively assign this band to D-mode of graphene substrate. Such band was not visible in the 532 nm-excited resonance Raman spectrum of graphene; however, activation of this band in SHINERS spectrum may be related with riboflavin adsorption induced tilt and distortion of graphene plane in the vicinity of Rf adsorption as predicted by DFT modeling (Figure 5). This observation highlights the ability to probe local perturbations by the SHINERS method.

## 4. Conclusions

In this paper, we provided detailed vibrational spectroscopy study of graphene-riboflavin surface. Several experimental vibrational spectroscopy methods, including ordinary Raman (riboflavin solution and powder spectra), resonance Raman (graphene spectra), graphene-enhanced Raman, and shell-isolated nanoparticle-enhanced Raman spectroscopies were employed to probe the structure and bonding of both single-layer graphene grown on copper and riboflavin adsorbate. We found that riboflavin adsorption induces the blue-shift of the 2D and G bands by 11 and 3 cm^−1^ respectively, indicating doping of the graphene. In addition, well-defined signatures of under-layered Cu_2_O oxide were obtained. We demonstrated that contrary to GERS approach, SHINERS method provides more detailed information about the interfaces; not only the riboflavin ring but also vibrational modes of ribityl chain and intense D-band of graphene were detected. DFT modeling suggested that this D-band may be activated because of riboflavin adsorption induced local perturbations (tilt and distortions) in the graphene structure. Our work highlighted the ability of SHINERS spectroscopy to probe the local structural perturbations in graphene.

## Figures and Tables

**Table 1:** Summary of calculations at B3LYP theory level for the G-Rf complex.

Parameters	6-311G(d,p)	def2-TZVP
d, Å	3.36	3.35
*E*_ad_, kcal/mol	−22.39	−33.35
Hirshfeld charge, e	0.12 (G) ^1^	0.11 (G) ^1^
	−0.12 (Rf) ^1^	−0.11 (Rf) ^1^
*E*_fund_, eV	2.39 (G-Rf) ^1^	2.31 (G-Rf) ^1^
	2.77 (Rf) ^2^	3.43 (Rf) ^2^

^1^ Optimized in G-Rf complex. ^2^ Optimized in a gas phase. Abbreviations: *E*_ad_, adsorption energy; *d*, the average distance between planes of graphene and the Flavin ring moiety; *E*_fund_, fundamental energy gap (*E*_LUMO_−*E*_HOMO_).

**Table 2:** Electronic transition wavelengths (*λ*), energies (*E*), oscillator strengths (*f*), and corresponding orbitals of G-Rf complex and Rf in gas phase-optimized form calculated at TD-DFT B3LYP/6-311G(d,p).

G-Rf Complex			Rf (Optimized in a Gas Phase)		
*λ*, nm (Orbitals)	*E*, eV	*f*	*λ*, nm (Orbitals)	*E*, eV	*f*
651 (270 ^b^↔271 ^a^) ^1^	1.904	0.0015	608 (99↔100) ^1^	2.037	0.0003
639 (269 ^b^↔271 ^a^)	1.940	0.0022	457 (98↔100)	2.707	0.1900
433 (268 ^a,b^↔271 ^a^)	2.860	0.0007	402 (97↔100)	3.078	0.0495
428 (267 ^b^↔271 ^a^)	2.893	0.0143	394 ((93,94)↔100)	3.145	0.0043

^1^ Corresponds to HOMO-LUMO orbitals. ^a^ Located at Rf. ^b^ Located at G. ↔ denotes the electronic transition.

**Table 3:** Assignments of vibrational bands observed in SHINERS and GERS study of graphene-riboflavin surface.

Peak Position (cm^−1^)	Raman Spectroscopy Method	Assignment ^1^
2675 vs	RRS	2D band; graphene
1590 m/1592 vw	RRS/SHINERS	G band; graphene
1626 w	GERS	Ring I and II stretch; Rf
1536 br, w/1525 br,w	GERS/SHINERS	Ring I, II, and III stretch; Rf
1457 s	SHINERS	CH_3_ deformation + Ring I, II stretch + Ribityl bend; Rf
1411 w/1407 w	GERS/SHINERS	CH_3_ bend; Rf
1351 w/1349 vs	GERS/SHINERS	C2-N3-C4 symmetric stretch + Ring I, II, III stretch; Rf
1321 s	SHINERS	D band; graphene
1206 w	SHINERS	Ring I breathing + Ring II, III stretch; Rf
1158 w	SHINERS	Ring I, II, III stretch + C2-N3-C4 symmetric stretch; Rf
1048 s	SHINERS	Ring I, II, III stretch; Rf
981 s	SHINERS	C-C + C-O stretch + COH deformation; Rf ribityl ^2^
930 m	SHINERS	C-C + C-N stretch + CH2 rock; Rf ribityl ^2^
808 w	SHINERS	N-C=O anti-symmetric bend + Ring I, II, III in-plane deformation; Rf
740 s	SHINERS	Ring I in-plane bending; Rf
710 w	SHINERS	Ring I, II, III in-plane deformation; Rf
640 w/626 w	GERS-RRS/SHINERS	Cu_2_O
559 w	SHINERS	N-C=O symmetric stretch + Ring I, II, III in-plane deformation; Rf
413 vw	GERS-RRS	Cu_2_O
219 m	GERS-RRS	Cu_2_O
151 w	GERS-RRS	Cu_2_O

^1^ Based on reference [58]. ^2^ Based on DFT calculations of G-Rf complex. Abbreviations: GERS, graphene-enhanced Raman scattering; SHINERS, shell-isolated nanoparticle-enhanced Raman scattering; RRS, resonance Raman scattering; Rf, riboflavin; vs, very strong; s, strong; m, middle; w, weak; vw, very weak; br, broad.

## Data Availability

The data presented in this article are available within this article.
